# Giving meaning to internalized violence throughout life by older adults living in rural areas

**DOI:** 10.1590/0034-7167-2023-0163

**Published:** 2024-06-17

**Authors:** Aline Balandis Costa, Maria Aparecida Salci, Francielle Renata Danielli Martins Marques, Vanessa Denardi Antoniassi Baldissera, Lígia Carreira

**Affiliations:** I Universidade Estadual do Norte do Paraná. Bandeirantes, Paraná, Brazil; II Universidade Estadual de Maringá. Maringá, Paraná, Brazil

**Keywords:** Elder Abuse, Aged, Violence, Rural Population, Gender-Based Violence, Abuso de Ancianos, Anciano, Violencia, Medio Rural, Violencia de Género

## Abstract

**Objectives:**

to understand the meanings of violence internalized throughout life by older adults living in rural areas.

**Methods:**

a qualitative study, anchored in the Symbolic Interactionism theoretical framework and the Grounded Theory methodological framework in the constructivist aspect. Data collection occurred through individual interviews. Data were coded using the Atlas.ti software.

**Results:**

it was possible to identify that the context of rural areas strengthens patriarchy culture as well as contributing to violence silence and naturalization. It was also found that violence is a product of social inequality and gender inequality.

**Final Considerations:**

older adults living in rural areas internalized the violence suffered in a unique way, and this scenario’s specific aspects can increase intra-family abuse, as there is a patriarchal culture that promotes social and gender inequality.

## RESUMEN

**Objetivos:**

comprender los significados de la violencia internalizada a lo largo de la vida por personas mayores que viven en zonas rurales.

**Métodos:**

estudio cualitativo, anclado en el marco teórico del Interaccionismo Simbólico y el marco metodológico de la Teoría Fundamentada en el aspecto constructivista. La recolección de datos se produjo a través de entrevistas individuales. Los datos fueron codificados utilizando el software Atlas. ti.

**Resultados:**

se pudo identificar que el contexto del área rural fortalece la cultura del patriarcado, además de contribuir al silencio y naturalización de la violencia. También se encontró que la violencia es producto de la desigualdad social y de la desigualdad de género.

**Consideraciones Finales:**

los adultos mayores residentes de zonas rurales interiorizaron de manera singular la violencia sufrida, y las particularidades de este escenario pueden incrementar la violencia intrafamiliar, pues existe una cultura patriarcal que promueve la desigualdad social y de género.

## INTRODUCTION

Population aging is a reality in developing countries and has become a global challenge^(1)^. In 2000, in Brazil, there were around 14 million older adults, however, in 2020, this number reached 29 million and, in 2025, it is estimated that Brazil will reach the sixth largest population of older adults in the world^(1,2)^. The impact of the COVID-19 pandemic drastically affected life expectancy, with a decline of 1.3 years, however Brazil continues to have an increasingly aged population^(3)^.

The rapid aging of the Brazilian population could represent a serious problem if it is not accompanied by adequate public policies. The significant increase in older adults culminates in the emergence of new challenges, such as coping with elder abuse, which has been growing significantly in recent years, being recognized as a public health concern^(4,5)^.

It is estimated that 16% of people over 60 have suffered some type of violence, the most frequent form being psychological abuse (6.9%), and it is identified that the majority of violence is intra-family, with children being the main aggressors^(6)^. In Brazil, studies have documented the prevalence of elder abuse ranging from 10.1% to 21%^(7)^. According to the United Nations, these numbers are increasing considerably due to population aging, and could reach 320 million victims by 2050^(8)^. The presence of violence in older adults’ daily lives is notorious, but there is a lack of studies regarding elder abuse in rural areas. There is a lack of specific actions that take into account the subjectivities and peculiarities emerging from the historical, social, cultural and daily context inherent to this population, causing the total silencing of the phenomenon of violence within this area to echo^(9,10)^.

Moreover, current society maintains patriarchal values, also evident in rural societies, which favors the increase in forms of inequality in society and especially among women^(11,12)^.

Brazil has invested efforts to address the inequities that affect the rural population and, in particular, those arising from gender inequality, through investments in the implementation of policies aimed at women’s and rural populations’ health. However, it is still necessary to increase efforts to take actions capable of reducing this problem and mitigating the impacts of violence in rural areas^(13)^.

Considering the complexity of the factors involved in elder abuse, added to the rural population’s cultural, social and historical aspects as well as the need to understand, based on the various unique relationships, the way they signify and internalize their experiences in situations of violence throughout their lives, this study is necessary in order to give visibility to this complex phenomenon.

## OBJECTIVES

To understand the meanings of violence internalized throughout life by older adults living in rural areas.

## METHODS

### Ethical aspects

All current ethical precepts for research involving human beings were followed. The project was assessed by the Research Ethics Committee (REC), which requested an opinion from the Brazilian National Ethics and Research Committee (CONEP), which issued a favorable opinion on the research. All participants were informed about the objectives, method used in the research as well as information about voluntary participation and confidentiality regarding the data collected. All, without exception, spontaneously signed the Informed Consent Form (ICF). To guarantee the confidentiality of the information collected and participant identity preservation, pseudonyms were used.

### Theoretical-methodological framework

The study is based on the Symbolic Interactionism (SI) theoretical framework^(14)^ and the Grounded Theory (GT) methodological framework from a constructivist perspective^(15)^.

SI values interaction and has mechanisms for understanding the relationships between objects and people in situations experienced in a specific social context. With regard to giving meaning to elder abuse in rural areas, SI made it possible to understand the actions and interactions in relationships that build such a process and, thus, it was possible to determine the meaning of this phenomenon throughout life from older adults’ perspective in rural areas.

GT seeks to understand social phenomena based on the meanings of relationships and interactions between people, and the constructivist approach must establish the study phenomena priorities, data observation and the analysis generated, based on experiences shared with participants. It is assumed that researchers do not discover a hidden or true theory, but that knowledge is the result of a construction between researcher and participant. Thus, using GT in the constructivist aspect in this study is justified because it argues that participants’ meanings and expressions are constructions of reality and interpretative theorization and because it is a methodological framework that provides broad knowledge about little explored phenomena, which are based, above all, on constant interactions between people, as is the case of elder abuse in rural areas.

### Study design

This is a qualitative study, which seeks to work with the universe of meanings, values, beliefs and attitudes. To maintain methodological rigor, COnsolidated criteria for REporting Qualitative research (COREQ) was used as a support tool for research development.

### Methodological procedures

This study followed all GT precepts in the constructivist aspect.

### Study setting

The study location was a rural neighborhood of a small municipality in the state of Paraná that had a Family Health Strategy (FHS) team in its territory, with registration and monitoring of the population of that area. At the end of selection, the research consisted of six older adults. It is worth considering that, to reach these participants, 52 older adults living in rural areas and belonging to this FHS were screened.

### Data source

The study population was composed of older adults living in rural areas. Older adults aged 60 years or older, living in a rural neighborhood, registered with FHS, with cognition preserved through the Mini Mental State Examination (MMSE), which takes into account the minimum score required for each level of education, and evidence of possible situations of violence throughout life, were included.

MMSE is comprised of two sections that measure cognitive functions. The first section contains items that assess orientation, memory and attention, totaling 21 points; the second measures the ability to name, obey verbal and written commands, freely write a sentence and copy a complex drawing (polygons), totaling nine points. The total score is 30 points, based on dichotomous items. The 23/24 cut-off points are used to suggest cognitive impairment for literate older adults. After applying MMSE, to screen for violence, the instrument “elder abuse and mistreatment assessment” was used, available in the Primary Care Notebook 19, annex 13, page 178. This instrument contains 14 questions that permeate the types of violence, as each yes answer leads researchers to suspect some type of violence. Exclusion criteria were not eligible.

### Data collection and organization

Data were collected between November 2021 and May 2022, through individual interviews, guided by a question guide that was prepared according to the GT premises. Before application, it was assessed by three experts in GT and elder abuse research. The interviews were carried out at FHS, in a private room, with prior scheduling, depending on participants’ availability. They lasted 36 and 115 minutes, with the help of audio recording technologies and were transcribed in full. Data collection and analysis were carried out concomitantly, as foreseen by GT in the constructivist aspect. In this study, three sample groups emerged: older adults who did not live with their aggressor husband; older adults who lived with their aggressor children; older adults who live with their abusive husband.

### Data analysis

The coding and analysis techniques occurred in two stages: initial and focused coding^(15)^. The ATLAS ti. version 22.06.0 (license: L-2DE-AEA) was used as a tool to help organize and analyze data. Memos and diagrams were also used to elucidate and analyze the theoretical model.

The central phenomenon of this study was “Giving meaning to violence of older adults living in rural areas”, presented in three categories: Unveiling the forms of violence suffered; Unveiling the factors that keep rural older adults in situations of violence; Experiencing strategies for coping and breaking with the cycle of violence. The theoretical model was validated by experts in the study area.

## RESULTS

The meanings and symbols attributed to the violence suffered throughout their lives by older adults who live in rural areas are inherent to people who have gone through the process of violence and, throughout their lives, internalized feelings that caused the violence to be silenced or were strategies to break with this cycle were found.

To theorize about the meanings of violence internalized throughout life by older adults living in rural areas, this manuscript is divided into three categories.

### Category 1 – Unveiling the forms of violence suffered

**Figure 1:**
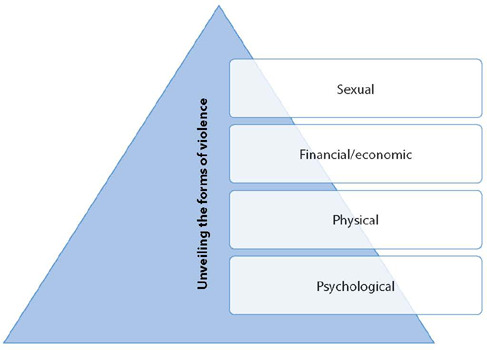
Category 1 diagram – Unveiling the forms of violence suffered

This category addresses all types of violence that were reported by older adults living in rural areas. All participants suffered intrafamily violence, with four types of violence reported (psychological, physical, financial and sexual). Psychological abuse was the most reported, followed by physical, financial and, finally, sexual. It is important to highlight that all participants mentioned associations of more than one type of violence suffered, with one of them reporting having suffered all types of violence (Figure 1).

Aggressors were spouses and/or children. When analyzing aggressors and the types of violence perpetrated by them, it is noted that the husbands committed the four types of violence, and children committed psychological and financial/economic abuse.

Psychological abuse occurs in many forms, some more explicit, others more veiled.

*I had a stiff back from working so much. After he ate, he changed his clothes, cleaned his shoes and left with a suitcase and said to me, “I’m going to leave, I’m wasting time with you here. I’m leaving so I don’t kill you, I’m wasting time with you, there’s a woman out there like an ant after me.” I stayed quiet for fear of him hitting me, because I was already stuck* [...]. *“The father leaves because his mother just doesn’t keep snakes, because she doesn’t know who the male is, because his mother leaves with everything that is male.” He spoke that way to the boys and left.* (Loiva)

*Wow, God, he used gave names, he invented things, he said that the children weren’t his, that I was seeing another man.* (Noeli)

*He said to me, “Where are you going, you don’t have the capacity”.* (Adélia)

Physical abuse was the second most frequent type reported by participants, and only one reported not having suffered any physical abuse throughout her life. It is perceived that physical abuse is concrete violence and is easily recognized by older adults. In these situations, victims are able to tell, in detail, what they experienced.

*You know, this* [she shows the scar on her arm] *was a stab he gave me. I was six months pregnant* [...] *he arrived, asked to heat water so he could take a shower, I turned to get the water and turned my back to him and he grabbed me by the hair, pulled me back and wanted to put the knife on my neck.* (Noeli)

*He came and slapped me in the face* [...] *and I had my oldest daughter on my lap, she was eight months old.* (Clementina)

*As he was hungry, one time he would grab me by the throat, another time he would squeeze me and I would turn all blue* [...] *he would arrive and start talking, talking, talking, and I would respond. Sometimes it would be about food and I would say, “You don’t bring food like you want me to without you bringing it into the house.” And he would come, grab me by the throat and the children would see, you know. And I didn’t do anything, right, at that time, I didn’t.* (Adélia)

*How many times did my boys take me to sleep next door, because he would use an iron bar to beat.* [...] *he came at me and grabbed me here* [shows his arm]*; he had big nails and tore everything up, look how it looked* [shows his arm with several scars]. *This is where you can see everything inside.* (Justina)

Without limits, disagreements and fights evolve into the execution of physical abuse which, in addition to leaving emotional and physical scars, also puts lives at risk.

*My eldest daughter died and I was eight months pregnant. My husband hit me and I fell down the stairs and she died. He punched me in the face and I ran from him and it was high, three steps, I stepped wrong and fell, as I hit my hand on the floor, my placenta detached.* (Loiva)

*Yes, you will* [...] *even if it’s in little pieces inside the suitcase, but you will. He tried to kill me, but God was more, he couldn’t.* (Loiva)

Financial abuse was the third most frequent type of violence that emerged from the data.

*Now, when I lived with him, I didn’t have a penny, everything went to him, I worked and he took the money in the afternoon. We bought something to eat and kept the rest. I took care, I gardened, I worked hard and he took the money.* (Noeli)

This violence happens more silently and often goes unnoticed by victims, who end up relating their lack of autonomy with money as something normal and occurring on a daily basis at the time.

*After I retired, I started to know about money, but before I took money and didn’t even see it, it was all with my husband, but we never lacked anything.* (Justina)

Financial elder abuse, when committed by children, has meaning attributed to motherhood itself, understood as an obligation to help children with their expenses.

*I give him a loan so he can finish his house. He also owed a debt to the house for materials to finish the house* [...]. *The car breaks down, the motorcycle breaks down, and I help him with everything.* (Luci)

In this context of financial abuse suffered, older adults had negative consequences regarding their personal needs and desires, experiencing financial restrictions, in addition to having their autonomy curtailed.

*It’s a lot to handle, if it were just me, I could. But now with the loans, bills, rent, medication, water, electricity, there is nothing left.* (Luci)

Sexual abuse was reported by two participants. However, this type of violence is the most difficult to report, as victims are ashamed to express their experiences. Statements show how the role of women and men is unequal, as women reported that, when they arrived home from work, in addition to taking care of the house, they needed to be “available” to meet their husbands’ wishes:

*Ah, he forced me sometimes, you know. He wouldn’t let me take medication, he said that women who take medication were whores and that they wanted to go out with another man. I was tired, I worked all day working on machines in the fields, I cleared the forest like a man, I worked the same way. He would arrive at night, I would bathe the children, cook food and he would just arrive, take a shower and eat. He didn’t help me at all and, when we went to bed, he started to bother me, there was no way, man, you know, you know, if we react, he gets angry, and I ended up accepting it.* (Noeli)

*My husband arrived at one o’clock, two o’clock in the morning, I don’t know where he came from. And he would torment me, I was there sleeping, sometimes, even without dinner, without anything.* (Adélia)

Another important aspect related to sexual abuse pointed out by older adults was the fact that they normalize abusive sexual relations between spouses, which are often understood as permissible for men in married life. It is also observed that aggressors have the ability to relativize, normalize and condition their partner to the violence practiced, making them believe that this condition is natural in couples’ intimate life. With the absence of understanding, women only understand and mean the sexually aggressive attitudes experienced many years later.

*Yes, for years. And another, when we got married, girl, he tore all my panties and I bled so much, we thought it had to be like that, right, that it was like that. I went through all this and, the next day, I felt bad and he looked at me and said it was normal, that every woman went through this and that she wasn’t soft like me.* (Adélia)

The sexual abuse suffered is linked to psychological abuse, with the coexistence of difficulties for victims in having credibility regarding the aggression they suffered. Important report made by an older adult, who lived with her aggressor for 17 years and had eight children, stated that she had never felt pleasure in sexual relations and also mentioned that many people doubted that she had suffered such violence.

*Because I had never kissed, hugged or anything. Think of something ridiculous like an animal, struggling. I had to do it by force, you know, without wanting to, without pleasure and anyway, I could have been in my period. I never felt pleasure; I didn’t even know what that was* [...] *and today, sometimes, there are people who ignore that, you know? And they say, “How did you manage to have all these kids? But to have a child, don’t you need to have pleasure?”.* (Adélia)

### Category 2 – Unveiling the factors that keep rural older adults in situations of violence

**Figure 2:**
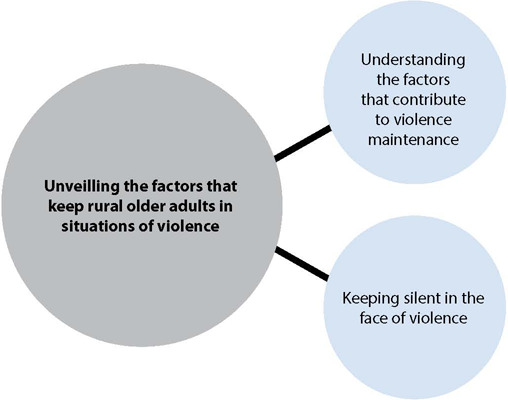
Category 2 diagram – Unveiling the factors that keep rural older adults in situations of violence

This category is made up of two subcategories: “Understanding the factors that contribute to violence maintenance”; and “Keeping silent in the face of violence” (Figure 2).

#### Subcategory 2.1 - Understanding the aspects that contribute to violence maintenance

Women gave meaning to the factors that contributed to greater vulnerability to violence over the years, such as the consumption of alcoholic beverages by aggressors, the presence of some mental disorder, weakened family ties with victims’ family and, at the same time, a concern by older adults in maintaining the established family unit, in addition to the patriarchal cultural values prevalent in the population living in rural areas.

Older adults’ reports showed that violence practice, mostly, occurred after using alcoholic beverages, and that, under the influence of this substance, they suffered physical and psychological abuse.

*Drinking, he liked to drink, but he wasn’t one for fighting and attacking, you know, he swore, he swore a lot.* (Luci)

*He always attacked me when he was drunk.* (Noeli)

Some cases of violence were associated with problems related to aggressors’ mental health. The husband’s violent acts were justified as a result of his mental disorder, which was also related to a history of aggressive behavior on the part of the spouse’s family, mentioning that the family had always been nervous people with explosive behavior.

*It’s been about 17 years since he got sick and became aggressive.* [...] *his whole family is* [violent], *but he attacked more because of the disease, he had a stroke and now it happened again and killed him.* (Justina)

Victims reported that aggressors, exclusively spouses, did not show feelings of affection and love for their family. They took a stance of moving away from their family of origin, showing that there are weakened family ties and that aggressors isolate and remove victims from life in society, thus preventing people around them from knowing about the attacks.

*No, he didn’t care about anything, he didn’t give love, he didn’t give affection, he didn’t give anything. For him, it was as if he were an animal.* (Adélia)

*No, they never knew* [victims’ parents] *because I got married here on a farm and, when I got married, he had a disagreement with my father and they fell out. And he* [husband] *took me and took me away from my family, he kept taking, taking, taking and to this day I don’t know if my family is alive, because I never had contact again. They don’t know my children; I’ve never seen my parents or my siblings again.* (Noeli)

Another aspect mentioned by older adults was the importance of maintaining the family unit as a social imposition, making it a necessary property in life in society. Victims keeping themselves in a violent relationship occurs in an attempt to preserve the established family, financial difficulties, concern about raising children, lack of support network and support to leave this relationship.

*I handle it because of the children. I tell them that it’s because of the children, we endured it, but it’s difficult, yes.* (Noeli)

*Today, there are services for women, women are more developed. Back then, you either handled it or you died.* (Loiva)

In addition to handling violence to preserve the family unit, the weight of patriarchal culture is also revealed, imposing regulations and customs. Therefore, many older adults remained subjugated to violence for many years due to fear or shame of separation and also due to socially imposed cultural beliefs and standards.

*My mother used to say, “A woman, if she leaves her husband, she becomes an odd job, she has to live with her husband until she dies”. I thought, “Oh my God, in this situation?”.* (Adélia)

*My mother-in-law asked me, in the agony of death, she said to me, “Noeli, I feel sorry for my son, because he will suffer a lot”. She asked and I think that’s why I put up with it, because she asked and I promised that I would never abandon her son, because she knew he would die in the gutter. In the situation she was in, I told her she could rest in peace and I would take care of her son, and she died, she rested.* (Noeli)

#### Subcategory 2.2 - Keeping silent in the face of violence

Victims of violence sometimes build mechanisms to silence the aggression they suffered, and thus remain in situations of violence, minimizing their own suffering. Some reasons led victims to silence and even naturalize the violence suffered, whether for fear of loneliness and helplessness or to avoid social embarrassment or for naturalizing the violence suffered as something inherent to “being an older adult” or “being a woman”.

Silencing for fear of helplessness or fear of breaking routine coexistence draws attention. Some victims show fear of facing aggressors, especially when aggressors are children, as they can cause disruption of the family relationship and lead to losses in meeting some care needs, inherent to age, and also not having anyone to count on, since many victims in this study live alone. Furthermore, there is also, in some cases, some emotional blackmail that children do to older adults, threatening to take their own lives and leaving older adults alone.

*She is my daughter, she is the only one who is by my side and helps me with anything* [...] *sometimes, she says things that I don’t like and I don’t answer, I stay quiet. Right here in this house, she often said that she was going to kill herself; she says she will take the rope and hang herself.* (Loiva)

Social embarrassment was also an important factor pointed out by older adults for the silencing, for many years, of the violence suffered. At the time and even today, women who are victims of violence are often criticized and judged by society as guilty instead of being protected.

*I thought to myself, “I’ve never seen anyone in my family fight, separate, now I, being my father’s first daughter to get married, would I separate?”. I thought, “I have to hold on”.* (Noeli)

Some women silenced violence by naturalizing it in the family environment, believing that, because they had not been physically attacked, it would be possible to overlook and naturally accept the lack of respect, defamation, among other acts of aggression.

*All couples have fights, but I don’t like swearing, I always tell him, “If he wants to talk, he can talk, but he doesn’t have to swear”.* (Clementina)

### Category 3 – Experiencing strategies for coping and breaking with the cycle of violence

**Figure 3:**
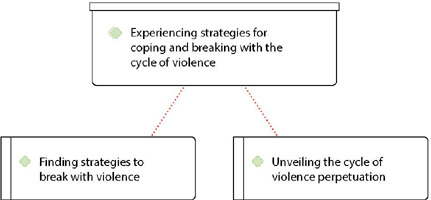
Category 3 diagram – Experiencing strategies for coping with and breaking with the cycle of violence

In this category, the main strategies adopted by victims to break with violence were listed, consisting of two subcategories: “Finding strategies to break with violence”; and “Unveiling the cycle of violence perpetuation” (Figure 3).

#### Subcategory 3.1 - Finding strategies to break with violence

Some strategies for coping with violence and breaking ties with aggressors were revealed, such as leaving the rural area and going to live in the urban area, acquiring financial autonomy and empowerment over their lives. Women were also revealed who maintained a bond with aggressors and sought strategies to live with violence, imposing limits and often physically confronting aggressors. It is important to highlight that, when the relationship with aggressors is marital, two participants broke the relationship. With three others, aggressors died and one sought strategies to live with aggressors to this day. When aggressors are children, victims found strategies to live with aggressors.

Female victims highlighted that leaving the rural area to live in the urban area made it possible to breaking with violence, as, in large urban centers, it was possible to expand employment opportunities as well as support to face such violence.

*I had all eight of my children here. And after a while, he said he wanted to go to São Paulo, that it was a good place to get a job. I didn’t know, but I went, and when I got there, I had the intention: this is where I’m going to stop all this. I thought, “I can’t be afraid, not of him, not of my mother, not of my father or of anyone, because I belong to no one”. And I told him, “Don’t touch on me anymore, because now I’m not that person anymore”. Then I discovered life in São Paulo, I went to the market, bought what I wanted and didn’t have to ask anyone. Today, I buy everything I want at the market.* (Adélia)

Empowerment and financial independence were also foundations for victims to break away from violence. One of them only achieved this financial autonomy after leaving the rural area.

*With seven children, I did this and he said to me, “Where are you going, you don’t have the capacity”. I told him, “I’m going to show you what I have and you’ll still enjoy my well-being”, and it was true. I raised my head and went near Ribeirão Preto, where I had never been. I didn’t know anyone there; I got a job and went to get my children and got my life together* [...] *I knew life like this, having the freedom to work, having my money and being able to buy things for my children, giving them what I wanted. Here, I worked on the farm, now, there, I worked at the mall, and it was this financial independence that made me give up.* (Adélia)

Older adults who maintained a bond with aggressors and were unable to break it found strategies to live with violence, imposing limits to continue living with aggressors. One of the strategies mentioned was to physically confront them and not show weakness.

*Many times, he would come at me with a knife or a shotgun and I would fight him. Every time he drank, he would go crazy, and since he lost some strength from being drunk, I was able to stand up to him.* (Noeli)

*Once, he slapped me, and then I got nervous and slapped him back. I told him not to hit me, not to put his hand on me. He said to me, “Because what are you going to do if I punch you and you won’t get up again?” I said, “That means I can’t get up anymore, because if I get up, I’ll kill you, even if I’m sleeping”, and he never hit me again.* (Clementina)

#### Subcategory 3.2 - Unveiling the cycle of violence perpetuation

It was possible to verify that violence perpetuation often occurs through transmission between generations, as a mechanism for perpetuating the problem is evident, suggesting that intra-family abuse is recurrent in homes where women and/or their partners were exposed to aggression, i.e., they grew up in a violent home. It was revealed that older adults who were victims of their partners also had experiences with intra-family abuse between their parents. The same was possible to see with aggressors’ history, i.e., aggressors also came from violent homes. Additionally, it was noticed that the daughters of older adult victims were more likely to suffer violence today.

Older adults, victims of violence, witnessed violence between their parents and family members in their homes, naturalizing violence. It was not clear whether these older adults suffered violence in their childhood, but they stated that their country’s marriage was always surrounded by violence.

*I got married and I always saw my father being aggressive towards my mother, you know, so I thought I had to be like my mother, humble, accept everything, get beaten up and not say anything. My father kicked me out on the street, you know, I was ten, 12, 14 years old; I had to go out and sleep at other people’s houses, at midnight, one o’clock in the morning. And so it was, I was revolting with all of this.* (Adélia)

It was possible to verify that aggressors also belonged to violent families.

*After I married him, I was horrified. I saw his brothers fighting, they almost killed each other, they drank, they went crazy, I went to a corner and thought that I had entered a family that I didn’t expect.* (Noeli)

*His father was very strange. His mother died at the age of 44, she died young and life for his mother and his father was very difficult, they fought a lot. I didn’t meet her. When I got married, she had been dead for six years, but she suffered a lot, her husband shot her and made her sleep under the coffee plants, sometimes he was raised was like that, right?* (Justina)

The daughter, who is currently an aggressor, was also a victim of physical abuse by her father, who was also an aggressor.

*She saw everything, she caught it too. He beat her a lot, if he ran into her on the street or in the yard, he would catch her outside and attack inside the house. It was like that, he was very bad*. (Loiva)

Another factor found in this research is that daughters of older adults who are victims of violence currently suffer violence in their marital relationships. It was realized that there is a cycle between generations and that, to break with it, it is quite challenging.

*The youngest lives with me. She got married, but her husband was very bad, he judged her and she returned to live with me. She and the children. Her whole body was burned, the marriage didn’t work out.* (Noeli)

*My daughters are married and one is separated, but they didn’t marry well; there is one who married a police officer and it was very*

*painful for her. She had a daughter who is now 15 years old and now she is with someone else and has had a little baby. But, for me, in my opinion, she doesn’t live well, no* [...] *one daughter has already caught her husband three times with another woman and she hasn’t given up on him. She says he is attentive and I look at my daughter, so beautiful and with an ugly man like that, toothless, bum, liar and I think, “How can someone be like that?”.* (Adélia)

## DISCUSSION

It is known that older adults constitute one of the most vulnerable groups to violence, the reasons for which range from social discrimination to the insufficiency of public policies to guarantee their rights^(16)^. In addition to this, there are rural populations and, consequently, aging in this context, which is demarcated by a set of economic, social, cultural, political and environmental aspects that connote greater social inequality and health vulnerabilities^(13,17)^.

Furthermore, violence is a product of social inequality and is also based on victims’ gender, as, according to the atlas of violence in 2017, more than 221 thousand women recorded physical aggression as a result of intra-family abuse, as this number is underestimated, as many do not report the violence they suffer. The dimension of violence associated with the position of subordination, devaluation and lack of recognition of women’s social role, even if in a veiled way, proves to be a fundamental determinant for the occurrence of violence in different contexts^(18)^.

The central phenomenon found in the research “Giving meaning to violence of older adults living in rural areas” supports the presence of gender-based violence in older adults living in rural areas and provides an expansion of knowledge on the subject, given that there is silence regarding this population. Although aging is an object of study with a vast production in specialized literature, studies aimed at understanding gender-based violence in rural areas, especially in Brazil, are still poorly quantified^(9,10,19)^.

It is possible to verify that the cultural, geographic, historical and social aspects of the older adult population in rural areas, as well as the patriarchal culture, added to low education, low income and being female, show the existence of the phenomenon of violence in rural areas, often silenced and naturalized by society. From this perspective, four types of violence were reported, the most frequent being psychological abuse, followed by physical, financial and, finally, sexual, with intra-family abuse being characterized as aggression by husband and children. However, the aggressors identified in the research were mostly male. These findings support the literature and demonstrate that the determining factors of elder abuse are multifaceted and are often associated with violence in the family environment^(4,11,20)^.

Family abuse, in general, is committed subtlety, making it difficult to identify, as it is often confused with everyday interpersonal stress, and, thus, mistreatment becomes naturalized^(21)^.

The aggressors’ profile was reported as being alcoholic or having some mental disorder and showing a certain emotional distance from their family, and always trying to keep victims away from their family of origin. Studies reveal that using alcohol and illicit drugs increases the risk of violence by up to three times and, combined with unemployment, are the most common factors for triggering intra-family abuse^(21,22)^. Psychiatric disorders, history of violence, abandonment, physical or sexual abuse in childhood are also factors found in the aggressors’ profile^(21,23)^.

In addition to the aggressors’ profile, two factors were identified as maintaining violence against older adults living in rural areas, which are the patriarchal values established in society and evidenced in rural areas and, consequently, the ideology of maintaining the family unit at all costs.

Structuring patriarchal values are associated with the serious recurrence of violence against women. However, the explanation of all forms of inequalities and oppression of women cannot be reduced to patriarchy, but it is evident that it is in the family environment that gender-based violence is more persistent and naturalized, as imposition of patriarchalism is rooted in these relationships^(11,12,24)^.

Faced with this naturalization rooted in society, older adults who live in rural areas silence the violence suffered in various ways, for fear of helplessness by family members or social embarrassment, because, many times, society blames victims for acts of violence and does not penalize aggressors and also because many of these older adults have naturalized this violence and believe it is inherent to family life^(24,25,26)^.

There are some strategies for coping and breaking with violence that were reported by older adults; however, it is known that the number of people who are able to face and break violence is very small in the country. The structure and culture for reporting are not widespread throughout the territory, and in rural areas, this difficulty is even greater, as it is necessary to consider the Brazilian territorial plurality, since, in rural areas, there are limitations, such as access difficulties due to the distance to the urban area and possession of mobile telephony and internet signal limitations^(10,27)^.

Another way to combat violence would be through informal support, consisting of community services, associations, shelters, support from religious and non-profit organizations, neighbors and people in the community to identify cases of violence. However, this is deficient in rural areas, as the rural exodus has contributed to increasingly reducing the number of residents in these spaces, which makes this support difficult^(10,27)^. It is noted that one of the alternatives pointed out by older adults participating in this research was to move to urban areas to face this problem.

Furthermore, female older adults also needed financial independence and empowerment to break away from the violence they suffered. Financial independence plays an important role in emancipating and empowering women so that they have opportunities to improve their living conditions, reduce inequalities and empower themselves to stop situations of violence^(26)^. It is necessary to improve women’s rights to access education and employment, as this will provide financial independence and, consequently, more autonomy over their lives^(26)^.

Another point revealed is violence perpetuation, i.e., the cycle between generations. The term used is “intergenerational transmission of domestic violence” (ITDV), which suggests that intrafamily violence has a higher incidence in homes where victims or aggressors have been exposed to violence^(28)^. In relation to intra-family abuse, it is necessary to consider the family relationship as a whole, taking into account the history of violence, because most aggressors lived in an environment permeated by different types of violence and began to reproduce what they witnessed throughout their lives^(28)^.

The literature shows that aggressors who have suffered aggression and violence in the past tend to repeat it in their family relationships through negative feedback, and this can also happen to victims who have lived in violent environments, as they tend to naturalize the violence suffered as part of their family environment^(28,29)^. Still in relation to ITDV, it is stated that experiences in a violent environment can lead to learning abusive behaviors as well as naturalizing the violence practiced and suffered. Violence cannot be justified with acts of violence, as there is the possibility for individuals to give new meaning to their negative experiences throughout their lives and, thus, employ coping mechanisms and breaking with the cycle of violence perpetuation^(29)^.

Older adults living in rural areas internalize and experience and give meaning to the violence suffered in a unique and particular way, showing that some aspects of the causality of violence are heightened in rural areas and that it is often necessary to leave this space to breaking with the cycle of violence. However, it is necessary to take into account the feelings of belonging to these spaces and thus subsidize resources so that the violence can be stopped without necessarily having older adults abandon their culture and origin. An alternative is implementing public health policies as well as engaging FHS in supporting victims of violence in terms of coping with and preventing this problem.

### Study limitations

As limitations, it is noted that this study covered a single Primary Health Care service and that the topic of violence generates a lot of prejudice and stigma. Therefore, the approach to participants is quite reduced and complex.

### Contributions to nursing, health, or public policy

This research contributes to advancing discussions about aging, after being victims of violence in rural areas and giving a voice to these people who are often silenced, as the great academic and theoretical production starts from gendered conceptions regarding aging in the rural environment. The urban context is always the scene of investigation and “where we talk” about being an older adult and also about public policies to disrupt and cope with the phenomenon of violence.

## FINAL CONSIDERATIONS

This study theorized the meanings of violence, throughout life, internalized by older adults living in rural areas. The results showed that older adults signify and internalize the violence suffered in a unique way, making it possible to understand that, in rural areas, the existence of intra-family abuse based on the culture of patriarchy as well as social and gender inequality has intensified.

With these data, it was possible to identify and understand aspects of this space, which can increase the intra-family abuse suffered by older adults. Furthermore, the results show the importance of advancing discussions about aging, after being victims of violence in rural areas and the need to give a voice to these people who are often silenced.

It is believed that it is necessary to reorganize Primary Health Care for older adults in rural areas, strengthening assistance in these areas with routine consultations as well as home visits, providing opportunities to strengthen access to formal protection networks and cope and breaking with violence. The motivation for intersectoral participation added to security, education and social assistance bodies is also important in these strategies.

Furthermore, it is necessary to train healthcare professionals who assist this population to identify cases of violence, carry out compulsory notification provided by law, in addition to supporting and guiding older adults in reporting and guaranteeing them, together with other professionals, safety to cope with the problem.
