# Supplementary material for: Giving meaning to internalized violence throughout life by older adults living in rural areas
Source: Rev Bras Enferm. 2024 Jun 17;77(3):e20230163. doi: 10.1590/0034-7167-2023-0163 (PMC11185073; doi:10.1590/0034-7167-2023-0163)
Supplement: Translation

# Translation: Significando a violência internalizada ao longo da vida por idosos que vivem na área rural

**DOI:** 10.1590/0034-7167-2023-0163pt

**Authors:** Aline Balandis Costa, Maria Aparecida Salci, Francielle Renata Danielli Martins Marques, Vanessa Denardi Antoniassi Baldissera, Lígia Carreira

**Affiliations:** I Universidade Estadual do Norte do Paraná. Bandeirantes, Paraná, Brasil; II Universidade Estadual de Maringá. Maringá, Paraná, Brasil

**Keywords:** Abuso de Idosos, Idoso, Violência, Zona Rural, Violência de Gênero

## Abstract

**Objetivos:**

compreender os significados da violência internalizados ao longo da vida por idosos que vivem na área rural.

**Métodos:**

estudo qualitativo, ancorado no referencial teórico do Interacionismo Simbólico e no referencial metodológico da Teoria Fundamentada nos Dados na vertente construtivista. A coleta de dados ocorreu por meio de entrevistas individuais. Os dados foram codificados com auxílio do *software* Atlas.ti.

**Resultados:**

foi possível identificar que o contexto da área rural fortalece a cultura do patriarcalismo, bem como contribui para que a violência seja silenciada e naturalizada. Constatou-se também que a violência é um produto da desigualdade social e da desigualdade de gênero.

**Considerações Finais:**

os idosos moradores da área rural internalizaram a violência sofrida de forma singular, e os aspectos próprios desse cenário podem potencializar a violência intrafamiliar, pois há uma cultura patriarcalista que promove a desigualdade social e de gênero.

## INTRODUÇÃO

O envelhecimento populacional é uma realidade dos países em desenvolvimento e tem se tornado um desafio mundial^(1)^. No ano de 2000, no Brasil, havia cerca de 14 milhões de idosos, porém, em 2020, esse número atingiu 29 milhões e, em 2025, estima-se que o Brasil alcance a sexta maior população de idosos do mundo^(1,2)^. O impacto da pandemia de COVID-19 afetou drasticamente a expectativa de vida, com declínio de 1,3 anos, contudo o Brasil continua apresentando uma população cada vez mais envelhecida^(3)^.

O rápido envelhecimento da população brasileira pode representar grave problema se não for acompanhado por políticas públicas adequadas. O significativo aumento de idosos culmina com o surgimento de novos desafios, como o enfrentamento da violência contra a pessoa idosa, que vem crescendo de forma expressiva nos últimos anos, sendo reconhecido como um problema de saúde pública^(4,5)^.

Estima-se que 16% das pessoas com mais de 60 anos já sofreram algum tipo de violência, sendo a forma mais frequente a violência psicológica (6,9%), e identifica-se que a maioria das violências é intrafamiliar, sendo o(a )filho(a) o principal agressor^(6)^. No Brasil, estudos documentaram prevalência da violência contra idosos variando de 10,1% a 21%^(7)^. De acordo com as Nações Unidas, esses números aumentam consideravelmente em razão do envelhecimento populacional, podendo chegar em 320 milhões de vítimas até o ano de 2050^(8)^. É notória a presença da violência no cotidiano da pessoa idosa, porém há escassez nos estudos referentes à violência contra a pessoa idosa na área rural. Nota-se a ausência de ações específicas que levam em conta as subjetividades e as peculiaridades emergentes do contexto histórico, social, cultural e do cotidiano inerente dessa população, fazendo que ecoe total silenciamento do fenômeno da violência dentro dessa área^(9,10)^.

Somado a isso, a sociedade atual mantém valores patriarcais, evidenciados também nas sociedades rurais, o que favorece o aumento das formas de desigualdade na sociedade e em especial sobre o gênero feminino^(11,12)^.

O Brasil tem investido esforços para enfrentar as iniquidades que afetam a população rural e, em especial, as oriundas da desigualdade de gênero, por meio de investimentos na implantação de políticas voltadas para a saúde da mulher e populações do campo. Porém, ainda é necessário aumentar os esforços para que se efetivem ações capazes de reduzir esse problema e mitigar os impactos da violência na área rural^(13)^.

Considerando a complexidade dos fatores envolvidos na violência contra a pessoa idosa, somado aos aspectos culturais, sociais e históricos da população rural, bem como a necessidade de compreender, a partir das diversas relações singulares, a forma como eles significam e internalizam suas vivências nas situações de violência, ao longo da vida, este estudo faz-se necessário, a fim de dar visibilidade a esse fenômeno complexo.

## OBJETIVOS

Compreender sobre os significados da violência internalizados ao longo da vida por idosos que vivem na área rural.

## MÉTODOS

### Aspectos éticos

Todos os preceitos éticos vigentes para pesquisas que envolvem seres humanos foram seguidos. O projeto foi apreciado pelo Comitê de Ética e Pesquisa (CEP), que solicitou parecer do Comitê Nacional de Ética e Pesquisa (CONEP), que emitiu parecer favorável à pesquisa. Todos os participantes foram esclarecidos quanto aos objetivos, método utilizado na pesquisa, bem como às informações sobre a participação voluntária e sigilo sobre os dados coletados. Todos, sem exceção, assinaram espontaneamente o Termo de Consentimento Livre e Esclarecido (TCLE). Para garantir o sigilo das informações coletadas e a preservação da identidade dos participantes, foram utilizados pseudônimos.

### Referencial teórico-metodológico

O estudo está alicerçado pelo referencial teórico do Interacionismo Simbólico (IS)^(14)^ e pelo referencial metodológico da Teoria Fundamentada nos Dados (TFD) na perspectiva construtivista^(15)^.

O IS valoriza a interação e possui mecanismos de compreensão das relações entre os objetos e as pessoas em situações vivenciadas em um contexto social específico. No que diz respeito ao significado da violência contra o idoso da área rural, o IS possibilitou a compreensão das ações e interações nas relações que constroem tal processo e, assim, foi possível determinar o significado desse fenômeno ao longo da vida na perspectiva do idoso da área rural.

A TFD busca a compreensão dos fenômenos sociais a partir dos significados das relações e interações entre as pessoas, sendo que a abordagem construtivista deve estabelecer as prioridades dos fenômenos do estudo, a observação dos dados e a análise gerada, a partir das experiências compartilhadas com os participantes. Parte-se do pressuposto de que o pesquisador não descobre uma teoria escondida ou verdadeira, mas que o conhecimento é fruto de uma construção entre pesquisador e participante. Assim, a utilização da TFD na vertente construtivista neste estudo justifica-se porque defende que os significados e as expressões dos participantes são construções da realidade e teorização interpretativa e por ser um referencial metodológico que proporciona conhecimento amplo sobre fenômenos pouco explorados, que se baseiam, sobretudo, nas interações constantes entre as pessoas, como é o caso da violência contra o idoso na área rural.

### Tipo de estudo

Estudo de abordagem qualitativa, que busca trabalhar com o universo de significados, valores, crenças e atitudes. Para manter o rigor metodológico, foi utilizado o protocolo *COnsolidated criteria for REporting Qualitative research* (COREQ) como ferramenta de apoio para o desenvolvimento da pesquisa.

### Procedimentos metodológicos

Seguiu todos os preceitos da TFD na vertente construtivista.

### Cenário do estudo

O local do estudo foi um bairro rural de um município de pequeno porte do estado do Paraná que possuía em seu território uma equipe da Estratégia Saúde da Família (ESF), com cadastro e acompanhamento da população daquela área adscrito. Ao final da seleção, a pesquisa foi composta por seis idosas. Vale considerar que, para alcançar essas participantes, foram rastreados 52 idosos moradores da área rural e pertencentes a essa ESF.

### Fonte de dados

A população do estudo foi composta por idosos residentes na área rural. Os critérios de inclusão adotados foram ter idade igual ou maior de 60 anos, morador do bairro rural, ser cadastrado na ESF, apresentar cognição preservada por meio do Mini Exame do Estado Mental (MEEM), que leva em conta a pontuação mínima necessária para cada grau de escolaridade, e apresentar indícios de possíveis situações de violência ao longo da vida.

O MEEM é composto por duas seções que medem funções cognitivas. A primeira seção contém itens que avaliam orientação, memória e atenção, totalizando 21 pontos; a segunda mede a capacidade de nomeação, de obediência a um comando verbal e a um escrito, de redação livre de uma sentença e de cópia de um desenho complexo (polígonos), perfazendo nove pontos. O escore total é de 30 pontos, baseados em itens dicotômicos. Os pontos de corte 23/24 são usados como sugestivos de déficit cognitivo para idosos alfabetizados. Após aplicação do MEEM, para o rastreio de violência, foi utilizado o instrumento “avaliação de violência e maus tratos contra a pessoa idosa”, disponível no Caderno de Atenção Básica nº. 19, anexo 13, página 178. Esse instrumento consta 14 perguntas que permeiam os tipos de violência, sendo que, a cada sim respondido, leva o pesquisador a suspeitar de algum tipo de violência. Não foram elegíveis os critérios de exclusão.

### Coleta e organização dos dados

Os dados foram coletados entre novembro de 2021 e maio de 2022, por meio de entrevistas individuais, guiadas por um roteiro de perguntas que foi elaborado segundo as premissas da TFD. Antes da aplicação, foi avaliado por três *experts* em pesquisa com TFD e violência contra a pessoa idosa. As entrevistas foram realizadas na ESF, em sala privativa, com agendamento prévio, conforme disponibilidade dos participantes. Tiveram duração de 36 e 115 minutos, com auxílio de tecnologias para a gravação dos áudios e foram transcritas na íntegra. A coleta de dados e a análise foram realizadas de forma concomitante, conforme prevê a TFD na vertente construtivista. Neste estudo, emergiram três grupos amostrais: idosas que não viviam com os agressores marido; idosas que conviviam com os agressores filhos(as); idosas que vivem com o agressor marido.

### Análise dos dados

As técnicas de codificação e de análise ocorreram em duas etapas: codificações inicial e focalizada^(15)^. Utilizou-se o *software* ATLAS ti., versão 22.06.0 (licença: L-2DE-AEA), como ferramenta de auxílio à organização e análise dos dados. Também foram utilizados memorandos e diagramas para a elucidação e análise do modelo teórico.

O fenômeno central do estudo foi “Significando a violência de idosos que vivem na área rural”, apresentado em três categorias: Desvelando as formas de violência sofrida; Desvelando os fatores que mantêm o idoso da área rural em situação de violência; Experienciando estratégias para enfrentamento e rompimento do ciclo de violência. O modelo teórico foi validado por *experts* na área do estudo.

## RESULTADOS

Os significados e a simbologia atribuídos à violência sofrida ao longo da vida por idosos que vivem na área rural são inerentes de pessoas que passaram pelo processo da violência e, ao longo da vida, internalizaram sentimentos que fizeram com que a violência fosse silenciada ou foram encontradas estratégias de ruptura desse ciclo.

Para teorizar sobre os significados da violência internalizados ao longo da vida por idosos que vivem na área rural, este manuscrito está dividido em três categorias.

### Categoria 1 – Desvelando as formas de violência sofrida

Essa categoria aborda todos os tipos de violência que foram relatados pelas idosas moradoras da área rural. Todas as participantes sofreram violência intrafamiliar, sendo relatados quatro tipos de violência (psicológica, física, financeira e sexual). A violência psicológica foi a mais relatada, seguida da física, financeira e, por último, sexual. É importante ressaltar que todas as participantes mencionaram associações de mais de um tipo de violência sofrida, sendo que uma delas relatou ter sofrido todos os tipos de violência (Figura 1).

**Figura 1:** Diagrama da categoria 1 – Desvelando as formas de violência sofrida

O vínculo com os agressores foi de cônjuge e/ou filhos(as). Ao analisar os agressores e os tipos de violência perpetradas pelos mesmos, nota-se que os maridos praticaram os quatro tipos de violência, e os(as) filhos(as) cometeram as violências psicológica e financeira/econômica.

A violência psicológica acontece de muitas formas, algumas mais explícitas outras mais veladas.

*Eu estava com a coluna travada de tanto trabalhar. Depois que ele comeu, ele trocou de roupa, limpou o sapato e saiu com uma mala e falou assim para mim: “Eu vou embora, eu estou perdendo tempo com você aqui. Eu vou embora para não te matar, estou perdendo tempo com você, lá fora tem mulher igual formiga atrás de mim”. Eu fiquei quieta com medo dele me bater, porque eu estava travada já* [...]. *“*O *pai vai embora porque sua mãe só não fica com cobra, porque não sabe quem é o macho, porque sua mãe sai com tudo que é homem”. Ele falou desse jeito para os meninos e foi embora.* (Loiva)

*Nossa, Deus, ele colocava cada nome, ele inventava as coisas, falava que os filhos não eram dele, que eu andava com outro homem.* (Noeli)

*Ele falou para mim: “Para onde você vai, você não tem capacidade”.* (Adélia)

A violência física foi o segundo tipo mais frequente relatado pelas participantes, e apenas uma relatou não ter sofrido nenhuma violência física ao longo da vida. É percebido que a violência física é a violência concreta e de fácil reconhecimento pela idosa. Nessas situações, as vítimas conseguem contar, com detalhes, o que viveram.

*Sabe, isso aqui* [ela mostra a cicatriz no braço] *foi uma facada que ele me deu. Eu estava grávida de seis meses* [...] *ele chegou, pediu para esquentar água para ele tomar banho, eu virei para pegar a água e dei as costas para ele e ele me pegou pelos cabelos, puxou para trás e queria passar a faca no meu pescoço.* (Noeli)

*Ele veio e deu um tapa na minha cara* [...] *e eu estava com a minha filha mais velha no colo, ela tinha oito meses.* (Clementina)

*Passando fome, uma hora ele me pegava pela garganta, outra vez me apertava e eu ficava toda roxa* [...] *ele chegava e começava a falar, falar, falar, e eu respondia. Às vezes, era sobre comida e eu falava: “Você não traz comida como que você quer que eu faça sem você trazer para dentro de casa”. E ele vinha, me pegava pela garganta e os filhos via, sabe. E eu como não tinha atitude, né, naquela época, eu não tinha.* (Adélia)

*Quantas vezes os meus meninos me levavam para dormir lá no vizinho, porque ele pegava barra de ferro para bater.* [...] *ele veio em mim e pegou aqui* [mostrou o braço]; *ele estava com as unhas grandes e rasgou tudo, olha como ficou* [mostra o braço com várias cicatrizes]. *Isso aqui ficou dando para ver tudo dentro.* (Justina)

Sem limites, os desentendimentos e as brigas evoluem para a execução da violência física que, além deixar cicatrizes emocionais e físicas, também colocam em risco a vida.

*A minha filha mais velha morreu eu estava grávida de oito meses. Meu marido me bateu e eu cai da escada e ela morreu. Ele deu um soco na minha cara e eu fui correr dele e era alto, três degraus, pisei em falso e cai, conforme eu bati a mão no chão, deu aquele arranque e descolou a placenta.* (Loiva)

*Você vai, sim* [...] *nem que for aos pedacinhos dentro da mala, mas você vai. Ele tentou me matar, mas Deus foi mais, ele não conseguiu.* (Loiva)

A violência financeira foi o terceiro tipo mais frequente de violência, que emergiu dos dados.

*Agora, quando eu morava com ele, eu não tinha um tostão, tudo ficava com ele, eu trabalhava e ele pegava o dinheiro a tarde. A gente comprava coisa de comer e ficava com ele o resto. Eu carpia, fazia horta eu trabalhava duro e ele pegava o dinheiro.* (Noeli)

Essa violência acontece de forma mais silenciosa e, muitas vezes, passa despercebida pela vítima, que acaba relacionando a sua não autonomia com o dinheiro como algo normal e cotidiano da época.

*Depois que eu aposentei que eu comecei a conhecer dinheiro, mas, antes eu pegava dinheiro e nem via, ficava tudo com meu marido, mas nunca faltou nada para nós.* (Justina)

A violência financeira contra a idosa, quando cometida por filhos, obteve significado atribuído à própria maternidade, compreendida como uma obrigação de ajudar o filho com suas despesas.

*Eu faço empréstimo para ele para ele terminar a casa dele. Ele fez também dívida na casa de materiais para terminar a casa* [...]. *O carro quebra, moto quebra, e eu que ajudo ele em tudo.* (Luci)

Nesse contexto da violência financeira sofrida, as idosas tiveram consequências negativas no que se referem às suas necessidades pessoais e desejos, passando por restrições financeiras, além de ter sua autonomia cerceada.

*É muita conta, se fosse só eu, dava. Mas, agora com os empréstimos, as contas, aluguel, remédio, água, luz, não sobra nada.* (Luci)

A violência sexual foi relatada por duas participantes. No entanto, esse tipo de violência é o mais difícil de ser relatado, pois a vítima tem vergonha de expressar suas vivências. As falas mostram como o papel da mulher e do homem é desigual, pois as mulheres relataram que, ao chegar do trabalho, além dos cuidados com a casa, precisavam estar “disponíveis” para atender aos desejos dos maridos:

*Ah, ele forçava eu às vezes, sabe. Ele não deixava eu tomar remédio, ele falava que mulher que toma remédio era puta e que queria sair com outro homem. Eu estava cansada, trabalhava o dia inteiro batendo máquina na roça, roçava mato igual homem, trabalhava igual. Chegava de noite, eu ia lavar as crianças, fazer comida e ele só chegava, tomava banho e comia. Não me ajudava em nada e, quando a gente ia deitar, ele começava a me incomodar, não tinha jeito, homem, sabe, né, se a gente reage, fica bravo e sem querer eu aceitava.* (Noeli)

*Meu marido chegava uma hora, duas horas da manhã, não sei de onde vinha. E ele ia atormentar, eu estava ali dormindo, às vezes, até sem janta, sem nada.* (Adélia)

Outro aspecto importante relacionado à violência sexual apontado pelas idosas foi o fato de elas normalizarem as relações sexuais abusivas dos cônjuges, muitas vezes compreendidas como permissíveis ao homem na vida conjugal. Observa-se ainda que o agressor tem a capacidade de relativizar, normalizar e condicionar a parceira à violência praticada, fazendo-a acreditar que essa condição é natural na vida íntima entre casais. Com a ausência de entendimento, a mulher só compreende e significa as atitudes agressivas sexualmente vividas muitos anos depois.

*Sim, por anos. E outra, quando a gente casou, menina do céu, ele rasgou toda a minha calcinha e eu sangrei tanto, a gente achava que tinha que ser assim, né, que era assim mesmo. Passei tudo isso e, no outro dia, eu fiquei mal e ele olhou para mim e disse que era normal, que toda mulher passava por isso e que não era mole igual eu.* (Adélia)

A violência sexual sofrida é atrelada à violência psicológica, coexistindo ainda uma dificuldade que a vítima tem de ter credibilidade quanto à agressão que sofreu. Relato importante feito por uma idosa, que viveu com o agressor por 17 anos e teve oito filhos, afirmou nunca ter sentido prazer nas relações sexuais e menciona ainda que muitas pessoas duvidavam que ela tivesse sofrido tal violência.

*Porque eu nunca tinha beijado, abraçado e nada. Pensa em uma coisa ridícula igual um animal, aos trancos e barrancos. Tinha que fazer na marra, sabe, sem querer, sem prazer e de qualquer jeito, podia estar menstruada. Nunca senti prazer; eu nem sabia o que era isso* [...] *e hoje, às vezes, tem pessoas que ignoram isso, sabe? E, falam: “Como que você conseguiu ter esse monte de filho? Ué, mas para ter filho, não precisa ter prazer?”.* (Adélia)

### Categoria 2 – Desvelando os fatores que mantêm o idoso da área rural em situações de violência

Essa categoria está composta por duas subcategorias: “Compreendendo os aspectos que contribuem para a manutenção da violência”; e “Silenciando-se diante da violência” (Figura 2).

**Figura 2:** Diagrama da categoria 2 – Desvelando os fatores que mantêm o idoso da área rural em situações de violência

#### Subcategoria 2.1 - Compreendendo os aspectos que contribuem para a manutenção da violência

As mulheres significaram os fatores que contribuíram para maior vulnerabilidade à violência ao longo dos anos, como o consumo de bebida alcoólica pelo agressor, a presença de algum transtorno mental, os vínculos familiares fragilizados com a família da vítima e, ao mesmo tempo, uma preocupação por parte da idosa na manutenção da unidade familiar constituída, além dos valores culturais patriarcais predominantes na população que vive em área rural.

Os relatos das idosas mostraram que as práticas da violência, majoritariamente, aconteciam após o uso de bebida alcoólica, e que, sob o efeito dessa substância, sofriam violência física e psicológica.

*Bebida, gostava de beber, mas ele não era de brigar e fazer agressão, sabe, ele xingava, xingava bastante.* (Luci)

*Ele me agredia sempre bêbado.* (Noeli)

Alguns casos de violência foram associados a problemas relacionados à saúde mental do agressor. Justificavam-se os atos violentos do marido em decorrência do transtorno mental, o que também esteve relacionado a um histórico de comportamento agressivo dos familiares do cônjuge, mencionando que a família sempre foi de pessoas nervosas e com comportamentos explosivos.

*Faz uns 17 anos que ele ficou doente e ficou agressivo.* [...] *a família toda dele é* [violenta], *mas ele atacou mais por causa da doença, deu derrame e agora deu de novo e matou ele.* (Justina)

As vítimas relatavam que os agressores, exclusivamente cônjuge, não apresentavam sentimentos de carinho e amor pela família. Elas assumiam uma postura de se afastarem de sua família de origem, evidenciando que há vínculos familiares fragilizados e que o agressor isola e afasta a vítima da vida em sociedade para, assim, evitar que as pessoas ao redor tenham conhecimento das agressões.

*Não, ele não ligava para nada, não dava amor, não dava carinho, não dava nada. Para ele, era como se fosse um animal.* (Adélia)

*Não, nunca souberam* [pais da vítima], *porque eu casei aqui em uma fazenda e, quando eu casei, ele teve uma desavença com o meu pai e eles se desentenderam. E ele* [marido] *me pegou e tirou de perto da minha família, foi tirando, tirando, tirando e até hoje eu não sei se a minha família é viva, nunca mais tive contato. Eles não conhecem meus filhos, nunca mais eu vi meus pais e nem meus irmãos.* (Noeli)

Outro aspecto mencionado pelas idosas foi a importância da manutenção da unidade familiar como uma imposição social, tornando uma propriedade necessária na vida em sociedade. A manutenção da vítima no relacionamento violento ocorre na tentativa de preservação da família constituída, dificuldades financeiras, preocupação com a criação dos filhos, ausência de rede de apoio e suporte para saírem dessa relação.

*Eu aguentei por causa dos filhos. Eu falo para eles que é por causa dos filhos, a gente aguentou, mas que é difícil, é.* (Noeli)

*Hoje, tem serviço para as mulheres, as mulheres são mais desenvolvidas. Naquela época, ou você aguentava ou morria.* (Loiva)

Além de aguentarem a violência para a preservação da unidade familiar, o peso da cultura patriarcal também é revelado, impondo normativas e costumes. Sendo assim, muitas idosas se mantiveram subjugadas à violência por longos anos por medo ou vergonha da separação e também pelas crenças e padrões culturais impostos socialmente.

*Minha mãe falava assim: “A mulher, se ela largar do marido ela vira biscate, tem que viver com o marido até morrer”. Eu pensava: “Meu Deus, nessa situação?”.* (Adélia)

*A minha sogra pediu para mim, na agonia da morte, ela falou para mim: “Noeli, eu tenho dó do meu filho, porque ele vai sofrer muito”. Ela pediu e acho que foi por isso que eu aguentei, porque ela pediu e eu prometi que eu nunca abandonaria o filho dela, porque ela sabia que ele ia morrer na sarjeta. Na situação que ela estava, eu falei para ela que podia descansar em paz que eu ia cuidar do filho dela, e ela morreu, descansou.* (Noeli)

#### Subcategoria 2.2 - Silenciando-se diante da violência

As vítimas de violência, por vezes, constroem mecanismos para silenciar a agressão sofrida, e assim, mantêm-se nas situações de violência, minimizando o seu próprio sofrimento. Alguns motivos levaram as vítimas a silenciar e até naturalizar a violência sofrida, seja por medo da solidão e do desamparo ou para evitar constrangimento social ou então por naturalizar a violência sofrida como algo inerente do “ser idoso” ou do “ser mulher”.

Chama a atenção o silenciamento por medo do desamparo ou medo de romper a convivência rotineira. Algumas vítimas demonstram receio de enfrentar o agressor, principalmente quando os agressores são filhos(as), pois podem causar rompimento da relação familiar e acarretar prejuízos no atendimento de algumas necessidades de cuidado, inerente da idade, e ainda de não ter com quem contar, visto que muitas vítimas deste estudo vivem sozinhas. Além disso, também há, em alguns casos, certa chantagem emocional que filhos(as) fazem com as idosas, ameaçando tirar sua própria vida e deixando a idosa sozinha.

*Ela é minha filha, é a única que está do lado e me ajuda com alguma coisa* [...] *às vezes, ela fala as coisas que não me agrada e eu não retruco, não, eu fico quieta. Aqui mesmo nessa casa, muitas vezes ela falou que vai se matar; fala que vai pegar a corda e se enforcar.* (Loiva)

O constrangimento social também foi um fator importante apontado pelas idosas para o silenciamento, por muitos anos, da violência sofrida. Na época e até nos dias de hoje, muitas vezes, as mulheres vítimas de violência são criticadas e julgadas pela sociedade como culpadas ao invés de serem protegidas.

*Eu pensava comigo: “Nunca vi ninguém da minha família brigar, separar, agora eu, sendo a primeira filha do meu pai a casar, eu ia separar?”. Pensei: “Tenho que aguentar”.* (Noeli)

Algumas mulheres silenciaram a violência por naturalizá-la no ambiente familiar, acreditando que, pelo fato de não terem sido agredidas fisicamente, seria possível relevar e aceitar com naturalidade a falta de respeito, a difamação, entre outros atos de agressão.

*Todos os casais têm briga, mas eu não gosto de palavrão, sempre falo para ele: “Se ele quiser falar, pode falar, mas não fala palavrão”.* (Clementina)

### Categoria 3 – Experienciando estratégias para enfrentamento e rompimento do ciclo de violência

Nessa categoria, foram elencadas as principais estratégias adotadas pelas vítimas para romper com a violência, composta por duas subcategorias: “Encontrando estratégias para a ruptura da violência”; e “Revelando o ciclo da perpetuação da violência” (Figura 3).

**Figura 3:** Diagrama da categoria 3 – Experienciando estratégias para o enfrentamento e rompimento do ciclo de violência

#### Subcategoria 3.1 - Encontrando estratégias para a ruptura da violência

Algumas estratégias para o enfrentamento da violência e rompimento de vínculo com o agressor foram reveladas, como sair da área rural e ir viver na área urbana, adquirir uma autonomia financeira e empoderamento sobre suas vidas. Foram reveladas também mulheres que mantinham vínculo com o agressor e buscaram estratégias para conviver com a violência, impondo limites e muitas vezes enfrentando o agressor fisicamente. É importante salientar que, quando o vínculo com o agressor é conjugal, duas participantes romperam o relacionamento. Com outras três, o agressor faleceu e uma buscou estratégias para conviver com o agressor até os dias de hoje. Quando o vínculo do agressor é filho(a), as vítimas encontraram estratégias para conviver com o(a) agressor(a).

As vítimas mulheres ressaltaram que a saída da área rural para viver na área urbana possibilitou o rompimento da violência, pois, em grandes centros urbanos, foi possível expandir as oportunidades de emprego, bem como de suporte para enfrentar tal violência.

*Eu tive todos os meus oito filhos aqui. E passou um tempo, ele disse que queria ir para São Paulo, que lá era bom para arrumar serviço. Eu não sabia, mas fui, e quando cheguei lá, eu tive a intenção: é aqui mesmo que eu vou parar com tudo isso. Eu pensei: “Eu não posso ter medo, nem dele, nem da minha mãe, nem do meu pai e nem de ninguém, porque eu não sou de ninguém”. E falei para ele: “Não coloca mais a mão em mim, porque agora eu não sou mais aquela, não”. Aí eu fui descobrir a vida em São Paulo, eu entrava no mercado, comprava o que eu queria e não tinha esse negócio de ficar perguntando para ninguém. Hoje, eu compro no mercado tudo que eu quero e que tenho vontade.* (Adélia)

O empoderamento e a independência financeira também foram alicerces para que as vítimas conseguissem romper com a violência. Uma delas só conseguiu essa autonomia financeira após a saída da área rural.

*Com sete filhos, eu fiz isso e ele falou para mim: “Onde você vai, você não tem capacidade”. Eu falei para ele: “Vou mostrar para você que eu tenho e você ainda vai desfrutar do meu bem-estar”, e foi verdadeiro. Eu ergui minha cabeça fui para perto de Ribeirão Preto, onde eu nunca tinha ido. Lá não conhecia ninguém, arrumei serviço e fui buscar meus filhos e arrumei minha vida* [...] *conheci a vida assim, de ter liberdade para trabalhar, de ter o meu dinheiro de poder comprar coisas para os meus filhos, dar o que eu queria. Aqui, eu trabalhava na roça, agora, lá, eu trabalhava no shopping,* e *foi essa independência financeira me fez dar um basta.* (Adélia)

Idosas que mantiveram o vínculo com o agressor e não conseguiram rompê-lo encontraram estratégias para conviver com a violência, impondo limites para continuar vivendo com o agressor. Uma das estratégias mencionadas foi confrontá-los fisicamente e não demonstrar fragilidade.

*Muitas vezes, ele vinha para cima de mim com faca, espingarda e eu enfrentava. Toda vez que ele bebia, ele ficava louco e, como ele perdia um pouco de força por estar bêbado, eu conseguia enfrentá-lo.* (Noeli)

*Uma vez, ele me deu o tapa, e aí eu fiquei nervosa e dei outro tapa nele. Eu falei para ele não bater em mim, não põe a mão em mim. Ele me falou: “Porque, o que você vai fazer se eu te der um murro você não levanta mais?”. Eu falei: “Isso me dá para não levantar mais, mesmo porque, se eu levantar, eu mato você, nem que for dormindo”, e nunca mais ele bateu em mim.* (Clementina)

#### Subcategoria 3.2 - Revelando o ciclo da perpetuação da violência

Foi possível constatar que a perpetuação da violência, muitas vezes, acontece na transmissão entre gerações, pois é evidenciado um mecanismo de perpetuação do problema, sugerindo que a violência intrafamiliar é recorrente em lares onde a mulher e/ou seu parceiro estiveram expostos à agressão, ou seja, cresceram em lar violento. Foi revelado que as idosas vítimas de seus parceiros também tiveram experiências com violência intrafamiliar entre seus pais. O mesmo foi possível perceber com o histórico dos agressores, ou seja, os agressores também eram oriundos de lares violentos. Somado a isso, foi percebido que as filhas das idosas vítimas tinham maior propensão de sofrerem violência na atualidade.

As idosas, vítimas de violência, presenciaram em seus lares violência entre seus pais e familiares, naturalizando a violência. Não ficou claro se essas idosas sofreram violência na infância, mas declararam que o casamento de seus país sempre foi rodeado de violência.

*Eu casei e eu sempre vi meu pai sendo agressivo com minha mãe, sabe, então eu achava que eu tinha que ser igual à minha mãe, humilde, aceitar tudo, apanhar e não falar nada. Meu pai me botava para rua, sabe, eu tinha dez, 12, 14 anos; eu tinha que sair e dormir na casa dos outros, meia-noite, uma hora da manhã. E, assim foi, eu fui revoltando com tudo isso.* (Adélia)

Foi possível verificar que os agressores também pertenciam a famílias violentas.

*Depois que eu casei com ele, eu fiquei horrorizada. Eu via os irmãos dele brigando, quase se matavam, bebiam, ficavam doidos, eu ia para um canto e ficava pensando que eu entrei em uma família que eu não esperava.* (Noeli)

*O pai dele era bem estranho. A mãe morreu com 44 anos, morreu nova e a vida da mãe mais o pai era bem difícil, eles brigavam muito, nossa. Eu não conheci ela. Quando eu casei, já tinha seis anos que ela tinha morrido, mas ela sofreu muito, o marido atirava nela e fazia ela dormir embaixo dos pés de café, às vezes é que a criação dele foi assim, né?* (Justina)

A filha que, atualmente, é agressora, também foi vítima de abusos físicos do pai, que também era agressor.

*Ela via tudo, ela apanhava também. Ele batia muito nela, se topasse com ela na rua ou no terreiro, ele já pegava ela lá fora e ia arrematar em mim dentro de casa. Era assim, ele era muito ruim*. (Loiva)

Outro fator encontrado nesta pesquisa é que as filhas das idosas vítimas de violência, atualmente, sofrem violência em suas relações conjugais. Foi percebido que existe um ciclo entre as gerações e que, para rompê-lo, é bastante desafiador.

*A caçula mora comigo. Ela casou, mas o marido era muito ruim, judiava dela e ela voltou a morar comigo. Ela e as crianças. Ela tem o corpo todo queimado, casamento não deu certo.* (Noeli)

*Minhas filhas são casadas e uma é separada, mas elas não casaram bem, não; tem uma que casou com policial e foi muito sofrido para ela. Ela teve uma filha que hoje tem 15 anos e agora ela está com outro e teve um nenenzinho. Só que, para mim, no meu pensar, ela não vive bem, não* [...] *uma filha já pegou o marido umas três vezes com outra mulher e ela não desisti dele. Ela fala que ele é atencioso e eu fico olhando para minha filha tão bonita e com um homem feio daquele desdentado, vagabundo, mentiroso e eu penso: “Gente, como que pode uma cabeça dessa?”.* (Adélia)

## DISCUSSÃO

Sabe-se que os idosos constituem um dos grupos mais vulneráveis à violência, cujos motivos vão desde a discriminação social até a insuficiência de políticas públicas para garantia dos seus direitos^(16)^. Somado a isso, têm-se as populações rurais e, consequentemente, o envelhecer nesse contexto, que é demarcado por um conjunto de aspectos de ordem econômica, social, cultural, política e ambiental que conotam maior desigualdade social e vulnerabilidades em saúde^(13,17)^.

Além disso, a violência é um produto da desigualdade social e também está alicerçado no gênero da vítima, pois, segundo o atlas da violência em 2017, mais de 221 mil mulheres registraram agressão física em decorrência da violência intrafamiliar, sendo que esse número é subestimado, pois muitas não denunciam as violências que sofrem. A dimensão da violência associada à posição de subordinação, à desvalorização e à falta de reconhecimento do papel social das mulheres, ainda que de maneira velada, mostra-se um determinante fundamental para a ocorrência da violência nos diferentes contextos^(18)^.

O fenômeno central encontrado na pesquisa “Significando a violência de idosos que vivem na área rural” sustenta a presença da violência de gênero em idosas moradoras da área rural e proporciona ampliação do conhecimento acerca da temática, visto que há o silenciamento sobre essa população. Apesar de o envelhecimento constituir um objeto de estudos com uma vasta produção na literatura especializada, os estudos voltados à compreensão da violência de gênero na área rural, principalmente no Brasil, ainda são pouco quantificados^(9,10,19)^.

É possível verificar que os aspectos culturais, geográficos, históricos e sociais da população idosa da área rural, bem como a cultura patriarcalista, somados à baixa escolaridade, baixa renda e ser do sexo feminino, mostram a existência do fenômeno da violência na área rural, muitas vezes silenciada e naturalizada pela sociedade. Nessa perspectiva, foram relatados quatro tipos de violência, sendo a mais frequente a violência psicológica, seguida da física, financeira e, por último, a sexual, sendo caracterizada como violência intrafamiliar agressões por marido e filhos(as). Entretanto, os agressores identificados na pesquisa foram majoritariamente do sexo masculino. Esses achados corroboram a literatura e demonstram que os fatores determinantes de violência contra a pessoa idosa são multifacetados e, frequentemente, associados com violência no ambiente familiar^(4,11,20)^.

A violência no ambiente familiar, de modo geral, é cometida de forma sútil, sendo difícil a sua identificação, pois, muitas vezes, é confundida com o estresse interpessoal cotidiano, e, assim, os maus-tratos tornam-se naturalizados^(21)^.

O perfil do agressor foi relatado como alcoolista ou que tinha algum transtorno mental e que apresentava certo distanciamento afetivo com a família, e sempre tentava afastar a vítima de sua família de origem. Estudos revelam que o uso de álcool e drogas ilícitas aumenta em até três vezes o risco de ocorrência de violência e, aliado ao desemprego, são fatores mais comuns para desencadear a violência intrafamiliar^(21,22)^. Transtornos psiquiátricos, histórico de violência, abandono, abuso físico ou sexual na infância também são fatores encontrados no perfil do agressor^(21,23)^.

Além do perfil do agressor, dois fatores foram identificados como mantenedores da violência contra as idosas que vivem na área rural, que são os valores patriarcais instituídos na sociedade e evidenciados na área rural e, consequentemente, a ideologia de manter a unidade familiar a todo custo.

Os valores patriarcais estruturantes estão associados à grave recorrência de violência contra as mulheres. Porém, não se reduz ao patriarcado a explicação de todas as formas de desigualdades e de opressão do gênero feminino, mas fica evidente que é no ambiente familiar que a violência de gênero é mais persistente e naturalizada, pois a imposição do patriarcalismo está enraizada nessas relações^(11,12,24)^.

Diante dessa naturalização enraizada na sociedade, as idosas que vivem na área rural silenciam a violência sofrida de várias formas, por medo do desamparo por parte dos familiares ou do constrangimento social, pois, muitas, às vezes, a sociedade culpa a vítima pelos atos de violência e não penaliza o agressor e também porque muitas dessas idosas naturalizaram essa violência e acreditam ser inerente do convívio familiar^(24,25,26)^.

Há algumas estratégias de enfrentamento e rompimento da violência que foram relatadas pelas idosas, porém sabe-se que o número de pessoas que conseguem enfrentar e romper a violência é muito pequeno no país. A estrutura e a cultura para a denúncia ou notificação não estão difundidas em todo o território, sendo que, na área rural, essa dificuldade é ainda maior, pois é necessário considerar a pluralidade territorial brasileira, visto que, na área rural, há limitações, como as dificuldades de acesso em razão da distância para área urbana e posse da telefonia móvel e as limitações de sinal de internet^(10,27)^.

Outra forma para o enfrentamento da violência seria por meio do apoio informal, constituído pelos serviços comunitários, associações, abrigos, apoio de organizações religiosas e sem fins lucrativos, vizinhos e pessoas da comunidade para identificação de casos de violência. Porém, isso é deficitário na área rural, pois o êxodo rural contribuiu para reduzir cada vez mais o número de moradores nesses espaços, o que dificulta esse apoio^(10,27)^. Nota-se que uma das alternativas apontadas por idosas participantes desta pesquisa foi mudar-se para o meio urbano para enfrentar tal problemática.

Agrupado a isso, as idosas mulheres também precisaram de independência financeira e empoderamento para conseguir romper com a violência sofrida. A independência financeira tem papel importante para emancipar e dar autonomia às mulheres, para que tenham oportunidades de melhorar sua condição de vida, reduzir as desigualdades e se empoderar para interromper as situações de violência^(26)^. É necessário melhorar os direitos das mulheres ao acesso à educação e emprego, pois isso proporcionará a independência financeira e, consequentemente, mais autonomia sobre sua vida^(26)^.

Outro ponto revelado é a perpetuação da violência, ou seja, o ciclo entre as gerações. O termo utilizado é “transmissão intergeracional de violência doméstica” (TIVD), que sugere que a violência intrafamiliar tem maior incidência em lares onde a vítima ou o agressor estiveram expostos à violência^(28)^. Em relação à violência intrafamiliar, é preciso considerar o relacionamento familiar como um todo, levando em consideração o histórico de violência, pois a maioria dos agressores viveu em um ambiente permeado por diversos tipos de violência e passa a reproduzir o que presenciou ao longo de sua vida^(28)^.

A literatura mostra que o agressor que sofreu agressões e violência no passado tende a repeti-la nos seus relacionamentos familiares através da retroalimentação negativa, e isso também pode acontecer com as vítimas que viveram em ambientes violentos, pois tendem a naturalizar a violência sofrida como parte do seu ambiente familiar^(28,29)^. Ainda em relação à TIVD, afirma-se que as experiências do ambiente violento podem conduzir a aprendizagem de comportamentos abusivos, bem como naturalizar a violência praticada e sofrida. A violência não pode ser justificada com atos de violência, pois há a possibilidade de os indivíduos darem um novo sentido às suas experiências negativas ao longo da vida e, assim, empregar mecanismos de enfrentamento e rompimento do ciclo de perpetuação da violência^(29)^.

As idosas moradoras da área rural internalizam vivenciam e significam a violência sofrida de forma singular e particular, mostrando que alguns aspectos da causalidade da violência são potencializados na área rural e que, muitas vezes, é preciso sair desse espaço para conseguir romper com o ciclo da violência. Porém, é necessário levar em consideração os sentimentos de pertença a esses espaços e, assim, subsidiar recursos para que o rompimento da violência aconteça sem necessariamente a idosa ter que abandonar sua cultura e origem. Uma alternativa é a efetivação das políticas públicas de saúde, bem como engajar a ESF no suporte às vítimas de violência no que tange ao enfrentamento e à prevenção desse agravo.

### Limitações do estudo

Como limitações, registra-se que este estudo teve abrangência de um único serviço de Atenção Primária à Saúde e que o tema violência gera muito preconceito e estigmas. Assim, a abordagem aos participantes é bastante reduzida e complexa.

### Contribuições para as áreas da enfermagem, saúde, ou política pública

Esta pesquisa contribui para o avanço nas discussões sobre o envelhecer, após serem vítima de violência na área rural e dar voz a essas pessoas que, muitas vezes, são silenciadas, pois a grande produção acadêmica e teórica parte de concepções generificadas quanto ao envelhecer no meio rural. O contexto urbano é sempre o cenário de investigação e de “onde se fala” sobre o ser idoso e também sobre políticas públicas para rompimento e enfrentamento do fenômeno da violência.

## CONSIDERAÇÕES FINAIS

Este estudo teorizou os significados da violência, ao longo da vida, internalizada por idosas que vivem na área rural. Os resultados evidenciaram que os idosos significam e internalizam a violência sofrida de forma singular, sendo possível compreender que, na área rural, intensificou-se a existência da violência intrafamiliar alicerçada na cultura do patriarcalismo, bem como na desigualdade social e de gênero.

Com esses dados, foi possível identificar e compreender aspectos desse espaço, que pode potencializar a violência intrafamiliar sofrida pelo idoso. Além disso, os resultados mostram a importância de avançar nas discussões sobre o envelhecer, após serem vítima de violência na área rural e a necessidade de dar voz a essas pessoas que, muitas vezes, são silenciadas.

Acredita-se que seja necessária a reorganização da Atenção Primária à Saúde para idosos da área rural, fortalecendo a assistência nessas áreas com consultas de rotina, bem como visitas domiciliares, oportunizando o fortalecimento ao acesso às redes de proteção formal e o enfrentamento e rompimento da violência. A motivação da participação intersetorial agregada aos órgãos de segurança, educação e assistência social também se faz importante nessas estratégias.

Além disso, é necessário capacitar os profissionais de saúde que atendem essa população para identificar os casos de violência, realizar a notificação compulsória prevista por lei, além de amparar e orientar o idoso na denúncia e garantir a eles, junto com outros profissionais, a segurança para o enfrentamento da problemática.
